# Aperture Ratio Improvement by Optimizing the Voltage Slope and Reverse Pulse in the Driving Waveform for Electrowetting Displays

**DOI:** 10.3390/mi10120862

**Published:** 2019-12-07

**Authors:** Zichuan Yi, Wenyong Feng, Li Wang, Liming Liu, Yue Lin, Wenyao He, Lingling Shui, Chongfu Zhang, Zhi Zhang, Guofu Zhou

**Affiliations:** 1 College of Electron and Information, University of Electronic Science and Technology of China, Zhongshan Institute, Zhongshan 528402, China; yizichuan@163.com (Z.Y.); liulmxps@126.com (L.L.); linyuesky@gmail.com (Y.L.); Shuill@m.scnu.edu.cn (L.S.); cfzhang@uestc.edu.cn (C.Z.); zz001@zsc.edu.cn (Z.Z.); 2 Institute of Electronic Paper Displays, South China Academy of Advanced Optoelectronics, South China Normal University, Guangzhou 510006, China; hwy956005579@outlook.com (W.H.); guofu.zhou@m.scnu.edu.cn (G.Z.); 3 Shenzhen Guohua Optoelectronics Tech. Co., Ltd., Shenzhen 518110, China

**Keywords:** electrowetting display, aperture ratio, driving waveform, hysteresis characteristic, ink distribution, response speed

## Abstract

Electrowetting display (EWD) performance is severely affected by ink distribution and charge trapping in pixel cells. Therefore, a multi structural driving waveform is proposed for improving the aperture ratio of EWDs. In this paper, the hysteresis characteristic (capacitance–voltage, C-V) curve of the EWD pixel is tested and analyzed for obtaining the driving voltage value at the inflection point of the driving waveform. In the composition of driving waveform, a voltage slope is designed for preventing ink dispersion and a reverse pulse is designed for releasing the trapped charge which is caused by hysteresis characteristic. Finally, the frequency and the duty cycle of the driving waveform are optimized for the max aperture ratio by a series of testing. The experimental results show that the proposed driving waveform can improve the ink dispersion behavior, and the aperture ratio of the EWD is about 8% higher than the conventional driving waveform. At the same time, the response speed of the driving waveform can satisfy the dynamic display in EWDs, which provides a new idea for the design of the EWD driving scheme.

## 1. Introduction

In recent years, low-power and reflective displays which are readable in the sun are favored by many scientific researchers [1]. As a kind of reflective display technology, the electrowetting display (EWD) is receiving more and more attention [2], and its initial products have been successfully applied to various fields [3]. However, a stable multi-grayscale video display of EWDs has still not been implemented.

In 1981, researchers proposed the EWD technology [4]. It can display grayscale by controlling the movement of the colored ink. In the past few decades, various driving systems and image processing methods based on multi-level grayscale dynamic EWDs have greatly improved display quality [5,6]. We have also proposed a driving system for multi-grayscale display in an EWD based on a field programmable gate array (FPGA) chip, which provides an important reference for optimizing the dynamic display of EWDs [7]. In the field of EWD driving waveform, the driving method based on amplitude modulation (AM) or pulse width modulation (PWM) has been proposed [8], and the driving voltage is used to control the shrinkage of the ink droplets. However, the ink in the EWD pixel shows a phenomenon of hysteresis, which has a negative effect on optical performance. The PWM compensates for the hysteresis to some extent, but the PWM means a reduction in response speed and it can reduce the effective frame for the video display, and the power consumption of the EWD grayscale display is also increased by using the PWM at the same time. In addition, a dynamic contact angle model is established based on the molecular dynamics theory; the shape evolution of droplets has been studied under different direct current (DC) driving conditions. In addition, the influence of liquid interface resonance on contact angle has also been studied under alternating current (AC) driving conditions [9]. However, there are only two types of driving waveforms for driving droplets: stepped DC driving signal and sinusoidal AC driving signal. The problem of ink splitting has been also proposed during coupling AC-common driving process [10]. A driving modulation scheme was used to improve the ink dispersion performance to a certain extent, but the ink showed instability during the driving process. In other driving modes, sinusoidal, bipolar, and single-pulse are used to drive electrowetting liquid lenses respectively, and it was found that the positive and negative polarities of the driving voltage have a significant effect on the charge trapping of liquid lenses [11]. This provides a possible direction for the optimization of the driving waveform for EWDs. Based on the EWD micro-space pixel units, it has been found that the ink motion shape can be controlled by applying driving voltage with different rising speed [12]. The EWD reflectivity was improved using this method, but the response time of the driving waveform became longer. 

In order to improve the aperture ratio of EWD pixels and the shrinkage form of the ink, the relationship among driving voltage, ink shape, pixel aperture ratio, and response time are studied in this paper. Then, a driving waveform is proposed for EWDs to improve the display effect according to the motion behavior of the ink and the structure characteristics of the EWD. Compared with the conventional driving waveform, the proposed driving waveform can improve the performance of charge trapping, ink dispersion, aperture ratio, and response time.

## 2. Principle of EWD Driving Waveform System

### 2.1. Electrowetting Equivalent Circuit Model

**Figure 1:**
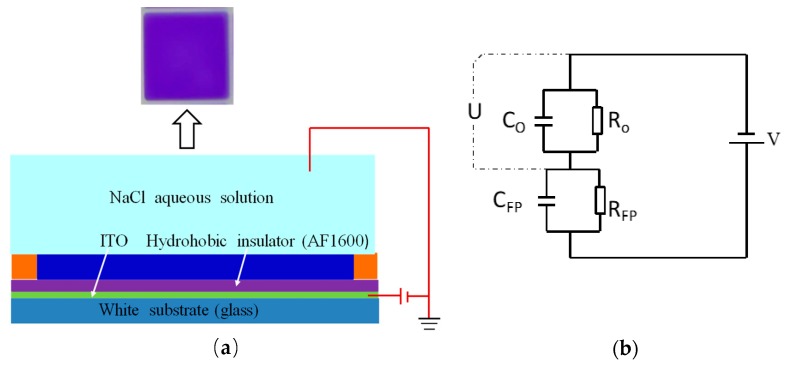
The electrowetting display (EWD) pixel structure and its equivalent circuit. (**a**) The EWD pixel structure without applied voltage; (**b**) simplified equivalent circuit diagram of an EWD pixel unit.

Grayscale display is realized in EWDs by applying voltage to control the movement of colored ink droplets. Its essence is an optical switch, which has excellent grayscale display characteristics. A typical EWD pixel structure is shown in Figure 1a. The orange part is the pixel wall, which can control the range of ink movement. From the top down, the ink is flat, and the state is also the lowest energy state.

The voltage is applied between the upper and lower plates which are made up of indium tin oxide (ITO). According to the Lippmann–Young equation, with the increase of the applied electric field, the surface tension among the insulating layer, ink film, and water can be increased, and the contact angle between the interfaces will also increase. The original balance is broken by the electric field force which is generated by the voltage difference; the ink is squeezed away by the water, and the water contacts the surface of the hydrophobic insulation layer, then, the white base plate is exposed. The aperture ratio (white area ratio) $W_{A}$ of a pixel is defined as Equation (1).

(1) $$W_{A}=1-(\frac{S_{\mathrm{ink}}(V)}{S_{\mathrm{pix}}})\times 100\%$$

In Equation (1), $S_{\mathrm{ink}}$ and $S_{\mathrm{pix}}$ are defined as the surface area of the ink in a single pixel and the surface area of the entire pixel, respectively, and V represents the voltage applied on the pixel in the EWD. In addition, the area of the pixel wall is ignored in the calculation of the aperture ratio. The displacement of the ink shrinkage is determined by the applied voltage [13,14]. Theoretically, the radius of ink shrinkage is directly related to the contact angle, and the contact angle θ follows the Lippmann–Young equation.

(2) $$\cos\theta =1-\frac{{\mathrm{CV}}^{2}}{2\gamma_{\mathrm{OW}}}$$

In Equation (2), C is the capacitance of a single pixel unit area. V is the voltage applied on the pixel unit, and $\gamma_{\mathrm{OW}}$ is the ink–water interfacial tension. The capacitance–voltage (C-V) curve provides an important parameter for driving EWDs and is an important basis for the design of driving waveform voltage.

For the equivalent circuit model of the micro-space pixel unit, the ink droplets are driven under an alternating electric field, and the ink and pixel wall in the same dielectric layer are treated as a combined loop. The photoresist material of the pixel wall has low conductivity, and the simplified equivalent circuit diagram is shown in Figure 1b [15]. $C_{0}$ is the ink capacitance, $R_{0}$ is the ink resistance, $C_{\mathrm{FP}}$ is the dielectric layer capacitance, $R_{\mathrm{FP}}$ is the dielectric layer resistance, and U is the effective voltage applied to the ink in the pixel. Based on the equivalent circuit model, the resistance, capacitance of the insulating layer, and ink follow Ohm’s law; then we can get their expressions of a single pixel.

Dielectric layer resistance is shown in Equation (3).

(3) $$R_{\mathrm{FP}}=\frac{d}{S\delta_{\mathrm{FP}}}$$

In Equation (3), d represents the thickness of the dielectric layer; S represents the area of a single pixel unit; $\delta_{\mathrm{FP}}$ represents the dielectric constant of the hydrophobic layer. The ink resistance is shown in Equation (4).

(4) $$R_{0}=\frac{H}{S\delta_{I}}$$

In Equation (4), H represents the thickness of the ink; $\delta_{I}$ represents the dielectric constant of the ink. The dielectric layer capacitance is shown in Equation (5).

(5) $$C_{\mathrm{FP}}=\varepsilon_{0}\delta_{\mathrm{FP}}S/d$$

In Equation (5), $\varepsilon_{0}$ is the vacuum dielectric constant. The ink capacitance $C_{I}$ is shown in Equation (6).

(6) $$C_{I}=\varepsilon_{0}\delta_{I}S/H$$

In Equation (6), $\delta_{I}$ represents the dielectric constant of the ink; $\varepsilon_{0}$ is the vacuum dielectric constant. According to Equations (3) and (4), the effective resistance R is shown in Equation (7).

(7) $$R=\frac{1}{S}(\frac{d}{\delta_{\mathrm{FP}}}+\frac{H}{\delta_{I}})$$

According to Equations (5) and (6), the single pixel cell effective capacitance C(H) is shown in Equation (8).

(8) $$C(H)=\varepsilon_{0}S(\frac{\delta_{\mathrm{FP}}}{H}+\frac{\delta_{I}}{d})$$

The critical starting voltage $V_{\mathrm{swith}}$ of a single EWD pixel unit can be estimated by Equation (9).

(9) $$V_{\mathrm{swith}}=\sqrt{\frac{2\pi^{2}\gamma_{\mathrm{ow}}}{C(H)S}}$$

The insulating film Teflon used in the EWD pixel cell structure has a correspondence relationship with the threshold voltage [16].

(10) $$\cos\theta_{\alpha }-\cos\theta_{0}=\frac{\delta \varepsilon_{0}U_{\mathrm{EW}}^{2}}{2d\gamma_{\mathrm{ow}}}$$

In Equation (10), $\theta_{\alpha }$ is the Lippmann contact angle; $\theta_{0}$ is the static contact angle; δ is the dielectric constant of a single pixel. The relationship between the applied voltage $U_{\mathrm{EW}}$ and the film thickness d of the insulating layer can be derived in Equation (11).

(11) $$U_{\mathrm{EW}}=\sqrt{\frac{2d\gamma_{\mathrm{ow}}}{\delta \varepsilon_{0}}(\cos\theta_{\alpha }-\cos\theta_{0})}\sim \sqrt{d}$$

The voltage of the insulating layer is proportional to the film thickness. The insulating layer is easily electrically broken when the film thickness of the insulating layer is too thin, so the parameters of the film thickness of the insulating layer should be considered in the design of the driving waveform.

### 2.2. Analysis of Ink Distribution in a Pixel 

**Figure 2:**
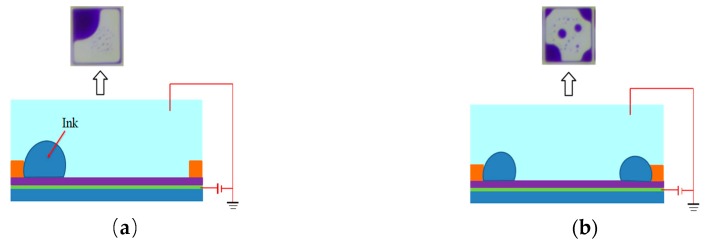
Ink film distribution state when the pixel is driven by the driving waveform. (**a**) Ink shrinks to one corner; (**b**) ink shrinks to four corners.

**Figure 3:**
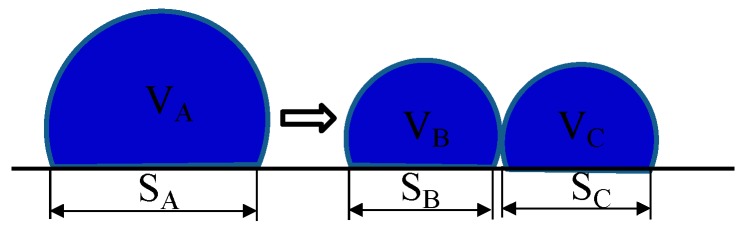
Ink splitting diagram.

In the process of driving an EWD, it is ideal to drive the ink to one corner of a pixel, as shown in Figure 2a, so we can get a max value of the aperture ratio at this time. However, once a conventional square driving waveform is applied, the ink may shrink to two or more corners, as shown in Figure 2b. The shape of the ink dispersion affects the EWD aperture ratio directly. As shown in Figure 3, one ink droplet A is divided into two droplets: B and C. Obviously, the covered area of the A droplet on the substrate S_A_ is smaller than the sum area of S_B_ and S_C_ and the aperture ratio becomes smaller when the ink is divided into two parts.

**Figure 4:**
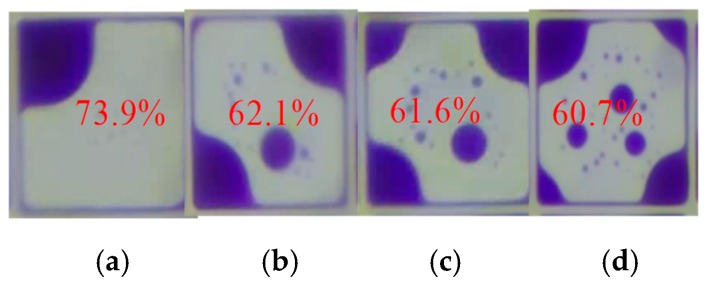
The ink distribution state and the corresponding aperture ratio with the action of the driving waveform. (**a**) The ink is all shrunk to one corner and its aperture ratio value is 73.9%. (**b**) The ink is dispersed into two parts and its aperture ratio value is 62.1%. (**c**) The ink is dispersed into three parts and its aperture ratio value is 61.6%. (**d**) The ink is dispersed into four parts and its aperture ratio value is 60.7%.

In an EWD pixel, the ink shape mainly has four shapes, as shown in Figure 4. Figure 4a is an ideal ink shape, the ink is all shrunk to one corner in a pixel. The maximum aperture ratio can be obtained, and the aperture ratio can reach 73.9%. However, if a conventional driving waveform is applied to the EWD panel, the ink may be dispersed into two parts as shown in Figure 4b, or dispersed into three parts as shown in Figure 4c, or dispersed into four parts as shown in Figure 4d. Obviously, the pixel can reach a max aperture ratio value when the ink distribution is stable. The aperture ratio values of different ink film distributions are shown as follows: 73.9%, 62.1%, 61.6%, and 60.7%, respectively. So, the greater the number of ink dispersions, the smaller the aperture ratio in the EWD pixel.

## 3. Driving Waveform Design

### 3.1. Testing System

**Figure 5:**
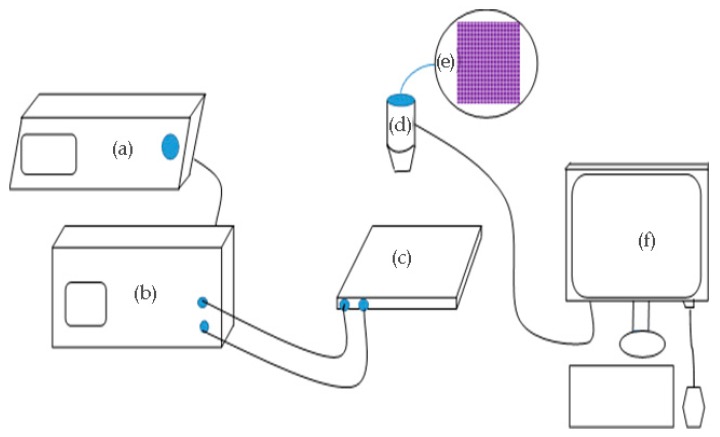
Optical testing system for EWDs. (**a**) AFG3052 arbitrary function generator; (**b**) ATA2022H high voltage amplifier; (**c**) testing board; (**d**) microscope; (**e**) pixels in an EWD; (**f**) computer.

In order to test the effects of the driving waveform, the experimental setup is designed as shown in Figure 5. In the tested EWD panel (2.7 inches diagonally), the size of a single pixel grid is 150 μm × 150 μm, and an entire panel contains 78,408 independent pixels. The width and height of the pixel wall are 15 μm and 5.6 μm, respectively, and the thickness of the insulating layer is 1 μm. The height between the ITO substrate and the upper cover in the pixel grid is 75 μm. The solvent C10H22 is used as a colored ink whose molecular concentration is 10 wt%, and the electrolyte is a sodium chloride solution whose concentration is 1.4 mol/L. The thickness of the spin-coated ITO glass substrate and the surface panel are 1.1 mm and 1.7 mm, respectively, and the impedance is 100 Ω/Sq. Teflon AF1600 is spin-coated on the surface of the glass substrate as a hydrophobic insulating layer, and the colored ink is injected into the pixel grid at a low speed (1 mm/s) by the grating filling method. Finally, the pixel unit is edge-sealed using a pressure sensitive element.

An Agitek AFG-3052C function generator and an Agitek ATA-2022H high voltage amplifier were used as driving devices. The driving waveform was edited by the PC and transmitted to an arbitrary function generator. The high voltage amplifier can amplify the driving waveform and output the driving voltage to the EWD. The process of ink breakage is recorded by a camera and the video format is saved, then the EWD aperture ratio data is calculated by an image processing software. The system is shown in Figure 5.

### 3.2. The Driving Waveform Structure

**Figure 6:**
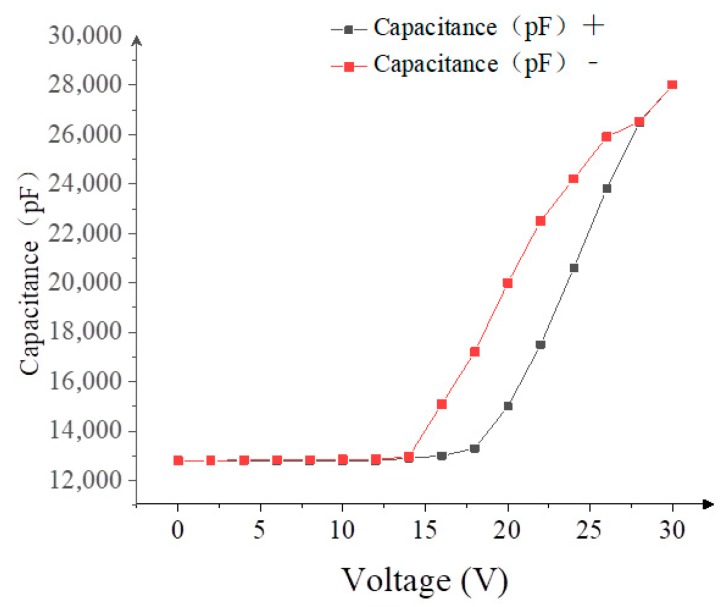
The relationship between the capacitance and the driving voltage in an EWD.

In the design of the driving waveform, the C-V curve provides important parameters for the driving waveform design. The C-V curve of a typical EWD panel is shown in Figure 6, which can be tested by the optical testing system in Figure 5. The driving voltage steps from 0 V to 30 V, and its speed is 4 V/s. At each step voltage, a high-speed camera is used to record the real-time image of the EWD pixel and calculate the aperture ratio. In Figure 6, the threshold voltage of the ink rupture whose value is 15 V can be observed clearly. The capacitance of the pixel increases sharply when the driving voltage is higher than the threshold voltage, and the amount of the charge at the EWD three-phase contact line increases rapidly. However, the optical response is not linear with the change of the driving voltage, which is a significant hysteresis between the rising and falling of the C-V curve. 

In addition, the threshold voltage for ink film reformation (red line) is lower than the threshold voltage for ink film dispersion (black line). According to the C-V curve, a large charge is rapidly accumulated in the hydrophobic insulator, which results in a sudden change of the electric field force which is likely to lead to ink dispersion when the capacitance value of the EWD pixel increases rapidly.

**Figure 7:**
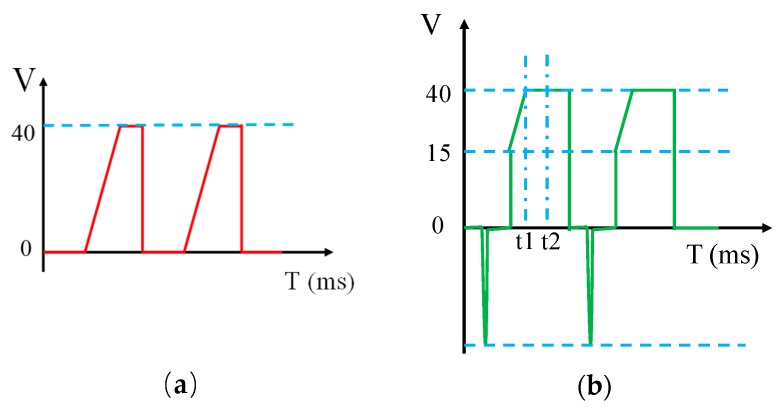
The structure of driving waveforms. (**a**) The driving waveform with a rising slope. (**b**) The proposed driving waveform in this paper.

In order to avoid ink dispersion, we designed a new driving waveform with three stages. In the first stage the driving voltage starts from 0 V, and a reverse electrode pulse voltage of several milliseconds is applied to remove the electric charge which is trapped in the hydrophobic insulating layer. Hence, the polarization and the hysteresis phenomenon are avoided. The step voltage in this stage is raised to the threshold voltage (15 V) which is shown in the C-V curve. At the second stage a driving voltage with a slope of 0.4 V/s is applied, and the ink film is prevented from being dispersed during the rupture of the ink film, so the aperture ratio of the EWD can be kept at a stable value. During the third stage the duty cycle of the driving waveform is adjusted to keep the driving voltage at a high level to maintain the shrinkage state of the ink. A driving waveform with a rising slope for improving the ink dispersion phenomenon has been proposed [12], as shown in Figure 7a. The voltage rising time of the driving waveform is t2–t1 slower than the proposed driving waveform in this paper, as shown in Figure 7b. 

The wetting mechanism of EWDs is related to the properties of ions in liquid solution. Because of defects in dielectric layer, ions in liquid can be bound by the dielectric layer easily, and the binding strength of the negative ion is greater than that of positive ion. Therefore, a large number of negative ions are bound by the dielectric layer when the positive voltage is applied. However, the negative ion cannot be released immediately when the applied voltage drops to 0 V, which results in hysteretic response of EWDs. According to the behavior of the ion which is trapped in dielectric layer, charge trapping in wetting dielectric layer can be controlled by the frequency of the driving waveform, reverse electrode driving mode and duty cycle coefficient (K) of the driving waveform.

## 4. Experimental Results and Discussion 

### 4.1. The Frequency of the Driving Waveform

The frequency of the driving waveform depends on two factors: the viscosity of the liquid and the charged ions in the hydrophobic insulating layer. The charged ion leads to the hysteresis effect of ink shrinkage and spread. In addition, the higher the ink viscosity, the higher the driving voltage or lower driving frequency. The conversion time of the charged ion can be shortened when the driving waveform has a high frequency, but there is not enough time to remove the charged ion trapped in the insulating layer, which leads to the accumulation of ions in the hydrophobic insulating layer; this is the main factor which leads to the hysteresis phenomenon of EWDs. Thus, the setting of the best frequency directly affects the performance of the driving waveform.

**Figure 8:**
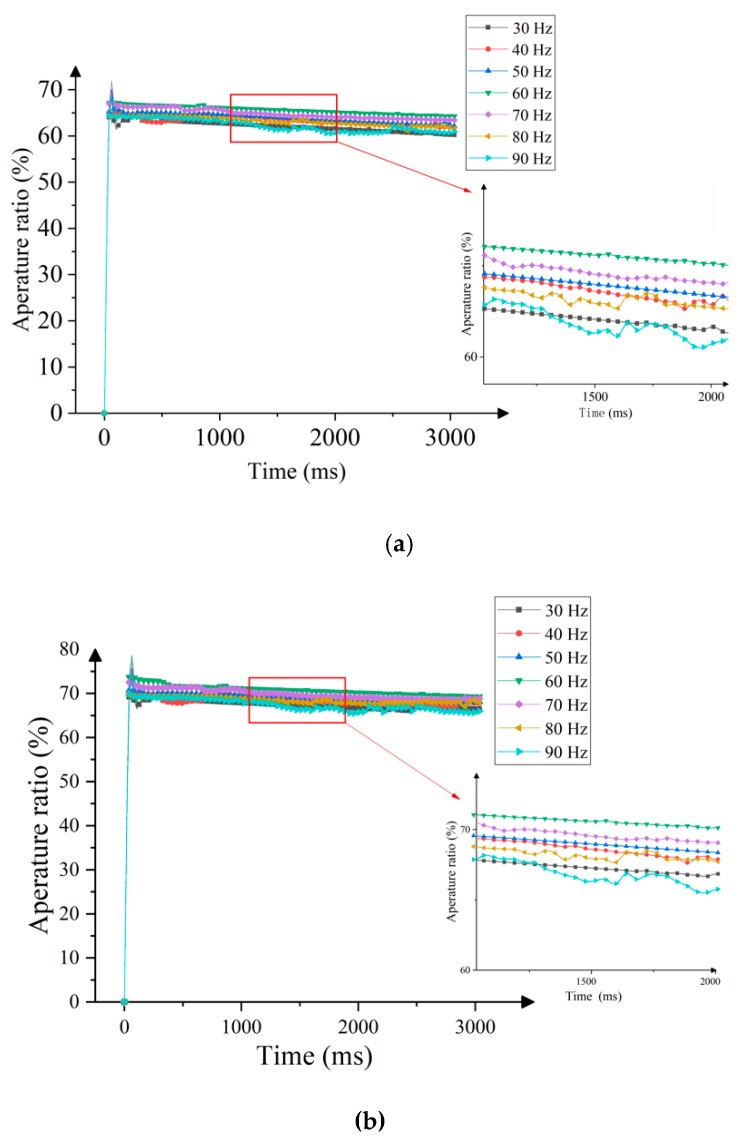
The relationship between the frequency and pixel aperture ratio. (**a**) Conventional driving waveform. (**b**) The proposed driving waveform in this paper.

In the testing process, the frequency of the driving waveform must higher than 25 Hz, because the flicker can be discerned by human eyes when the frequency is lower than 25 Hz [7]. Therefore, the conventional driving waveform and the proposed driving waveform in this paper are tested with different driving frequencies (30–90 Hz), which are higher than 25 Hz. The experimental results are shown in Figure 8. The aperture ratio is increased at the beginning, and then, it gradually decreases in the process of driving waveform frequency change from 30 Hz to 90 Hz; the highest aperture ratio is about 60 Hz. Hence, the change of ink shrinkage is related to the frequency of the driving waveform, which determines the release rate of charged ions. The aperture ratio value is smaller when the frequency is lower.

**Figure 9:**
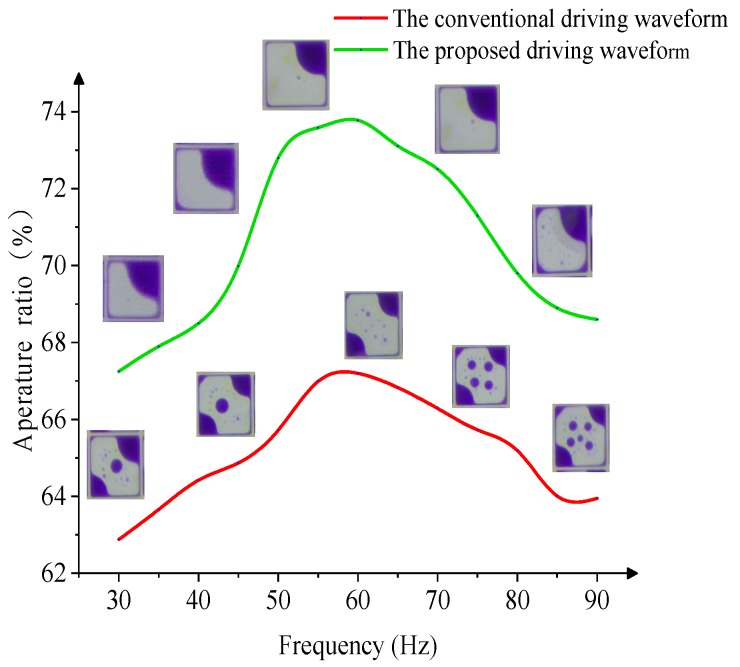
The relationship between aperture ratio (ink distribution) and the frequency of the driving waveform.

In addition, the ink motion is mainly affected by charged ions which bind in dielectric layer, ink viscosity, and friction. However, the ink aperture ratio fluctuates when the ink motion lags behind the conversion of the driving waveform, which can lead to the decrease of the average aperture ratio, as shown in Figure 9. The aperture ratio of the pixel and the number of charges bound in the dielectric layer are decreased when the frequency is increased gradually. At the same time, the charged ion accumulated at the three-phase contact line is decreased, but the change speed of the charged ion cannot keep up with the voltage polarity conversion. In addition, the oscillation amplitude of the ink can be decreased when the frequency of the driving waveform is increased. So, T ≈ 1/60 s is the key cycle for the stable shrinkage of the ink in the EWD, as shown in Figure 9.

### 4.2. The Duty Cycle of the Driving Waveform

**Figure 10:**
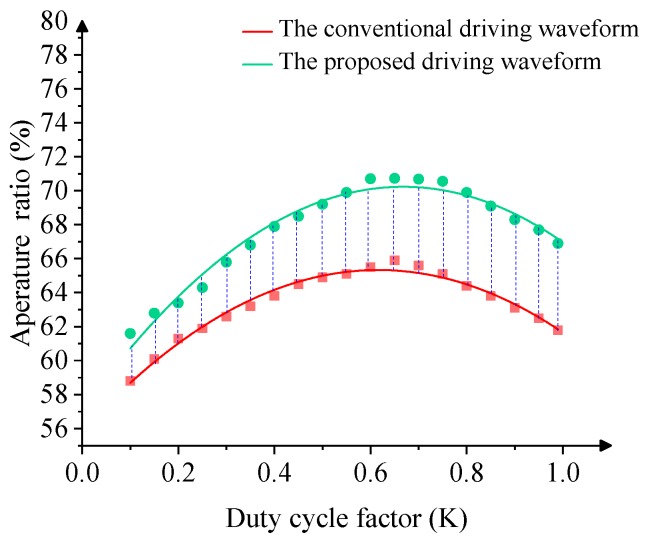
The relationship between the aperture ratio and the duty cycle in the driving waveform.

The aperture ratio of EWDs is determined by the voltage conversion frequency of the driving waveform. Nevertheless, the duty cycle of the driving waveform is another important factor for the performance of EWDs. In this paper, the experimental results show that the proposed driving waveform with a modulated duty cycle has better performance. Compared with the conventional driving waveform, the charged ion’s behavior can be better controlled and the pixel aperture ratio can be improved by optimizing the duty cycle. In Figure 10, with the same driving voltage and key period (T ≈ 1/60 s), the performance of the conventional driving waveform and the proposed driving waveform in this paper are compared in respect to the different duty cycle coefficients. Obviously, the two kinds of driving waveform have a maximum aperture ratio when the duty cycle coefficient is K ≈ 0.65. So, the duty cycle of the proposed driving waveform is set as 0.65.

### 4.3. The Performance of Driving Waveforms

The aperture ratio and the response time are important factors in the driving waveform of EWDs. However, a slower slope of voltage rising speed can achieve a maximum aperture ratio in EWDs. In other words, the slower the voltage rising slope, the longer the response time required, which affects the display frame rate seriously.

**Figure 11:**
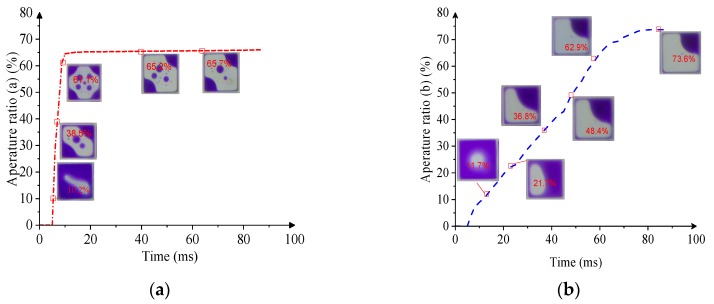

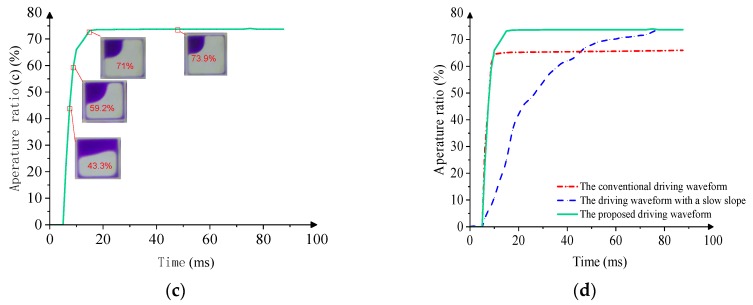
Change process of the EWD aperture ratio under driving waveforms. (**a**) Conventional driving waveform. (**b**) Driving waveform with a slow slope. (**c**) The proposed driving waveform in this paper. (**d**) Comparison of aperture ratio and response time among three driving waveforms.

As shown in Figure 11, the aperture ratio change trend of three driving waveforms are presented. Obviously, the shortest response time (8 ms) is achieved when the conventional driving waveform is applied, as shown in Figure 11a, but the ink is dispersed and the EWD aperture ratio is lowest. The response time becomes longer (65 ms) when a voltage slope is inserted into a driving waveform, as shown in Figure 11b; the ink is not dispersed and the aperture ratio of EWD increases significantly. In Figure 11c, the proposed driving waveform of this paper can achieve a shorter response time (15 ms). At the same time, the aperture ratio can also reach the maximum value (74%), which is about 8% higher than that of the conventional driving waveform. In Figure 11d, the proposed driving waveform can effectively control the shape of the ink, and a maximized aperture ratio is achieved. In order to obtain a clear comparison, parameter values among three kinds of driving waveforms are shown in Table 1.

## 5. Conclusions

In order to solve the problems of ink dispersion, hysteretic response, and low aperture ratio caused by the defect of the EWD dielectric layer, a driving waveform with a reverse electrode pulse and an optimized voltage slope was designed in this paper. A series of experiments were executed to test the driving waveform, specifically to include the pixel aperture ratio, frequency, and the duty cycle of the driving waveform. The results show that the aperture ratio can reach its largest value when the frequency of the driving waveform is about 60 Hz and duty cycle coefficient is 0.65. Compared with the conventional driving waveform, the aperture ratio increased by about 8%, and the ink steadily shrunk without dispersion. Hence, the display quality of EWDs was improved by optimizing the driving waveform.

## Figures and Tables

**Table 1:** Comparison of parameter values among different driving waveforms.

Driving Waveform	Conventional Driving Waveform [6,7]	The Driving Waveform with a Slope [12]	Proposed Driving Waveform
Aperture ratio	65.7%	73.6%	73.9%
Response time	8 ms	65 ms	15 ms
Shape of ink	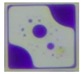	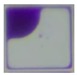	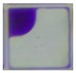
